# Psychometric properties of the Swedish version of the Parenting Concerns Questionnaire in parents with cancer

**DOI:** 10.2340/1651-226X.2024.40728

**Published:** 2024-07-22

**Authors:** Lisa Ljungman, Maria Romare Strandh, Niklas Gustafsson, Anna C. Muriel, Cynthia W. Moore, Pia Enebrink, Anna Wikman

**Affiliations:** a Department of Women’s and Children’s Health, Uppsala University, Uppsala, Sweden; b Centre for Women’s Mental Health during the Reproductive Lifespan (WOMHER), Uppsala University, Uppsala, Sweden; c Department of Psychosocial Oncology and Palliative Care, Dana Farber Cancer Institute, Boston, MA, USA; d Division of Child and Adolescent Psychiatry, Massachusetts General Hospital, Boston, MA, USA; e Department of Psychiatry, Harvard Medical School, Boston, MA, USA; f Department of Clinical Neuroscience, Karolinska Institutet, Stockholm, Sweden

**Keywords:** Oncology, parenting, distress, psychometrics, validation study

## Abstract

**Background and purpose:**

Parenting concerns can be a major source of distress for patients with cancer who are parents of dependent children; however, these are often not addressed in health care. The Parenting Concerns Questionnaire (PCQ) is an instrument designed to assess parents’ worries about the impact of cancer on their children and their ability to parent during this time. The Swedish version of the PCQ has, however, not been evaluated. This study therefore aimed to examine the psychometric properties of the PCQ in a sample of Swedish parents with cancer.

**Material and methods:**

A sample of 336 patients with cancer having dependent children (≤18 years) were included in a cross-sectional web-based survey. Participants completed questionnaires assessing parenting concerns, depression, anxiety, and stress symptoms (DASS); self-efficacy, family functioning (FAD-GF); and sociodemographic and clinical characteristics. Descriptive analyses, as well as reliability and validity analyses, were conducted followed by a confirmatory factor analysis of the factor structure proposed by the authors of the original version of the PCQ.

**Results:**

The majority were mothers (94.9%) with breast cancer (66.4%) aged 40–50 years (59.5%). The results showed evidence for convergent, criterion, and known group’s validity, but the original three-factor structure of the PCQ was not fully supported by confirmatory factor analysis.

**Interpretation:**

Evaluating parenting concerns may be an important step towards identifying patients who could benefit from targeted psychosocial interventions. However, the PCQ may require some further refinement to fully capture the breadth of parenting concerns in parents with cancer in different settings.

## Introduction

A large proportion of adults with cancer are parents of dependent children [1]. Parents with cancer must therefore not only deal with the demands of a cancer diagnosis and treatment but also manage the responsibilities associated with parenting. As such, parenthood has been recognized as a significant source of additional stress among parents with cancer [2–6], with single parents bearing an extra burden compared to those in a partnership [2,4]. Parenting concerns, defined as parents’ worries about the impact of cancer on their children and their ability to parent during this time, are a less studied area in psycho-oncology and are often not addressed in health care [6,7].

Previous studies have highlighted specific areas of parenting concerns including a fear of missing out and not being able to see their children grow up [2,6] and concerns about how to communicate with children about the cancer and how to handle the children’s emotional reactions [8,9]. Parenting concerns are higher among parents with incurable cancer [10], especially in relation to how their death will affect their children; parents feel a need to rush parenting, as they know that their time with their children is limited [6,11]. Studies also show that parental well-being is very much dependent on their children’s well-being [3,6,12,13]. Some parents struggle with feelings of being an inadequate parent [3,14], for example, due to having a hard time coping with the demands of the parental role [15]. Further, parenting competence is negatively affected by poor parental well-being, resulting in insufficient parenting strategies [16]. Some parents also worry about the competence of their co-parent to care for the children, both practically and emotionally, if they die [6]. Overall, parenting struggles can result in worry, depression, anxiety, and feelings of shame in parents with cancer but may also lead to poor family functioning [17]. In addition, high parenting concerns, low self-efficacy, and poor family functioning are associated with depression, anxiety, and stress among parents with cancer [3,18]. Hence, there is a strong link between parenting concerns and the well-being of parents, their children, and the entire family.

The *Parenting Concerns Questionnaire* (PCQ) was developed to assess parenting concerns relating to the emotional and practical impact of cancer on children and concerns relating to the co-parent in parents with cancer of any stage or type [10]. The PCQ was shown to have good reliability and moderate correlations, in the expected directions, with depression and anxiety, quality of life, and overall distress [10]. The psychometric properties of the PCQ have since been evaluated in German [19] and Portuguese [20] parents with cancer, as well as in American mothers with metastatic cancer [21]. Although these studies have largely supported the psychometric properties of the instrument, confirming the original three-factor structure, this was not supported in metastatic cancer, and an adapted version of the PCQ has now been developed for advanced cancer [22]. The aim of this study was to evaluate the psychometric properties of the Swedish version of the PCQ in parents with cancer who are caring for dependent children.

## Material and methods

### Study design and sample

Data were obtained from a longitudinal study of parenting concerns, family function, and psychological well-being in parents with cancer. For the purposes of the present study, cross-sectional data from the first assessment point in the longitudinal study were included in the analyses. Information about the study was distributed via a number of patient organizations and clinical contacts in Sweden, who in turn distributed the information to their members or patients through their own communication channels. In addition, information was spread using social media networks including Facebook and Instagram. Using these methods, it was possible to recruit a self-selected sample of 406 participants from all parts of Sweden. The participants were parents (aged 25–60 years) diagnosed with any type of cancer within the last 5 years who had at least one child aged 18 years or younger and were able to complete the survey in Swedish. Time since cancer diagnosis was defined as from the date of primary diagnosis or from the date of the last recurrence. As the PCQ includes items related to having a partner, not only a co-parent, an additional inclusion criterion was used for the purposes of the present analyses – that of having a partner. As such, 70 parents were excluded, resulting in a sample of 336 parents with cancer.

Data were collected using the web-based survey platform REDCap from 25th of January 2023 until 31st of May 2023. REDCap is a secure, web-based application designed to support data capture for research studies. Participants completed the survey anonymously but provided electronic informed consent and an e-mail address for subsequent follow-up assessments in the longitudinal study and remuneration for participation. Participants could choose to receive or decline a gift voucher for completing the survey at a value of 200 SEK. Ethical approval was obtained from the Swedish Ethical Review Authority (Dnr: 2022-03088-01).

### Translation of the PCQ

The original English version of the PCQ was translated into Swedish independently by two researchers who are native Swedish speakers fluent in English. The translated items were then compared and any differences resolved. The translated version was then backward translated to English by a native English speaker fluent in Swedish. The backward-translated version was compared with the original English version and discrepancies discussed both within the research group and with the authors of the original scale. These discussions resulted in only minor language adjustments to the final version of the Swedish PCQ.

### Measures

#### Background characteristics

Self-reported sociodemographic and clinical characteristics included participant’s age (in years), parenting role (mother, father), education level (less than university degree, university degree), number and age of children (youngest child, in years), cancer diagnosis (breast, central nervous system, gynecological, hematological, skin, lung, upper and lower gastrointestinal, urological, and other [grouped as other due to low numbers: head and neck cancer, thyroid cancer, sarcoma, and squamous cell cancer]), time since diagnosis (<1, 1–2, >2 years), cancer status (curable, incurable), self-reported history of mental health problems prior to cancer diagnosis (no, yes), and if experiencing a need for professional support for distress related to parenting and cancer (no, yes).

#### Parenting Concerns Questionnaire

The PCQ is a 15-item self-report measure of parental concerns among patients with cancer on three subscales: concerns about the emotional impact on their children, concerns about the practical impact on their children, and concerns about the co-parent/partner when there is one [10]. The subscales include five items each scored on a 5-point Likert scale as follows: (1) ‘Not at all concerned’, (2) ‘A little concerned’, (3) ‘Somewhat concerned’, (4) ‘Very concerned’ and (5) ‘Extremely concerned’. The mean-item total score and subscale scores range from 1 to 5, with higher scores indicating greater parental concerns.

#### Depression, anxiety and stress

The Depression Anxiety and Stress Scale-21 (DASS-21) was used to assess symptoms of depression, anxiety and stress on three subscales [23]. DASS-21 consists of 21 items scored on a 4-point Likert scale from 0 (did not apply to me at all) to 3 (applied to me very much or most of the time). The three subscales are scored separately and multiplied by 2, each with a total score ranging from 0 to 42, with higher scores indicating greater symptom severity.

#### Family functioning

Family functioning was assessed using the general functioning scale of the McMaster Family Assessment Device (FAD-GF) [24]. FAD-GF is 12-item measure of global family functioning with scores ranging from 1 to 4, where higher scores indicate worse family functioning. A score above two indicates unhealthy family functioning.

#### General self-efficacy

The General Self-Efficacy (GSE) scale was used to assess the strength of parents’ beliefs in their own ability to respond to novel or difficult situations and to deal with any associated obstacles or setbacks [25]. Total scores range from 10 to 40, with a higher score indicating greater self-efficacy.

### Statistical analyses

Descriptive statistics were used to present the participants’ characteristics and scores on the PCQ. Cronbach’s alpha coefficients were calculated to assess the internal consistency of the total scale and each of the three subscales. Corrected item-total correlations were calculated to test item discrimination, with higher correlations indicating greater consistency with the total scale. Values above 0.40 are considered satisfactory [26]. Pearson’s correlation coefficients were calculated to test convergent validity between the PCQ total and subscales and the DASS, FAD-GF, and the GSE scale. We anticipated more severe symptoms of depression, anxiety, and stress to be positively correlated with parenting concerns, and that greater parenting concerns would be associated with greater family dysfunction and lower self-efficacy scores. Independent samples *t*-tests, with Cohen’s d for effect sizes, were used to assess criterion validity by comparing scores on the PCQ total and subscales among those reporting using support services for parental distress (either having been offered support or having sought support from healthcare professionals) and known group’s validity by comparing parents with incurable cancer to those with curable cancer. Factorial validity was tested using confirmatory factor analysis with maximum likelihood estimation to test the original three-factor structure. Model fit was assessed using χ^2^ statistics, comparative fit index (CFI), and root mean square error of approximation (RMSEA). A significant χ^2^ test would indicate poor model fit; however, being sensitive to sample size, the ratio of χ^2^/*df* should be analyzed, where a ratio less than three is considered reasonably good. A CFI value >0.95 is representative of a well-fitting model, and a RMSEA value <0.06 indicates a good fit [27–29]. Analyses were conducted using IBM SPSS Statistics for Macintosh (Version 28.0) and R 4.3.3.

## Results

### Participant and PCQ characteristics

The characteristics of the 336 parents with cancer included in the study are presented in Table 1. The majority of parents were mothers (*n* = 319, 94.9%) aged 40–50 years (*n* = 200, 59.5%) with a university degree (*n* = 231, 68.7%). Breast cancer was the most common cancer diagnosis in the sample (*n* = 223, 66.4%), and nearly 1 in 5 participants had incurable cancer (*n* = 62, 18.5%). Time since diagnosis was evenly spread in the sample with over one-third of participants diagnosed less than a year prior to the survey (*n* = 123, 36.6%), over one-third within 1–2 years (*n* = 128, 38.1%), and the remainder more than 2 years earlier (*n* = 85, 25.3%). A history of self-reported mental health problems prior to the cancer diagnosis was prevalent (*n* = 123, 36.6%). The majority reported experiencing a need for professional support for distress experienced in relation to parenting and cancer (*n* = 192, 57.1%).

**Table 1:** Participant characteristics (*n* = 336).

Characteristics	Mean or n	SD or %
**Age (years)**	44.7	6.5
<40	70	28.8
40–50	200	59.5
>50	66	19.6
**Parent with cancer**		
Mother	319	94.9
Father	17	5.1
**Education level**		
Less than university degree	105	31.3
University degree	231	68.7
**Number of children (≤18 years)**	1.8	0.7
**Age of youngest child (years)**	10.3	5.1
**Cancer diagnosis**		
Breast cancer	223	66.4
Central nervous system (CNS) cancer	7	2.1
Gynecological cancer	26	7.7
Hematological cancer	16	4.8
Skin cancer	13	3.9
Lung cancer	4	1.2
Upper gastrointestinal cancer	9	2.7
Lower gastrointestinal cancer	26	7.7
Urological cancer	5	1.5
Other*	7	2.1
**Time since diagnosis (years)**		
<1	123	36.6
1–2	128	38.1
>2	85	25.3
**Cancer status**		
Curable	274	81.5
Incurable	62	18.5
**History of mental health problems (self-reported)**		
No	213	63.4
Yes	123	36.6
**Need for support for distress related to parenting and cancer**		
No	144	42.9
Yes	192	57.1

* Grouped as ‘other’ due to small numbers: head and neck cancer, thyroid cancer, sarcoma, and squamous cell cancer.

Descriptive statistics for the PCQ total score, subscales, and items are presented in Table 2. The mean score on the total PCQ was 2.27 (SD 0.79). Mean scores for the three subscales were: 2.52 (SD 0.99) for practical impact, 2.16 (SD 0.92) for emotional impact and 2.12 (SD 0.98) for the co-parent scale. Floor effects ranged from 15.2 to 64.3% and ceiling effects ranged from 1.5 to 13.7%. The largest floor (participants selecting the lowest response alternative, 1) and ceiling (participants selecting the highest response alternative, 5) effects were observed within the co-parent subscale (items 14 and 15, respectively).

**Table 2:** Descriptive statistics for the Parenting Concerns Questionnaire (*n* = 336).

PCQ	Mean	SD	80^th^ percentile	90^th^ percentile	95^th^ percentile	Floor (%)	Ceiling (%)	Corrected item-total correlation	Cronbach’s alpha
**Total score**	2.27	0.79	2.97	3.35	3.87				0.90
**Practical impact**	2.52	0.99	3.40	3.86	4.40				0.85
Item 1: My own mood, worries or emotions are affecting my children	2.87	1.19				15.2	8.6	0.67	
Item 3: My physical limits or low energy are affecting my children	2.57	1.27				28.0	6.8	0.67	
Item 5: I am not able to spend as much time with my children as I’d like	2.05	1.17				44.0	3.9	0.62	
Item 8: My illness is changing my children’s routine	2.74	1.31				20.8	12.5	0.71	
Item 10: Changes in my memory or attention are affecting my children	2.37	1.31				35.4	8.0	0.63	
**Emotional impact**	2.16	0.92	3.00	3.46	4.00				0.85
Item 2: My children are emotionally upset by my illness	2.62	1.26				22.6	8.0	0.73	
Item 4: My children are worried that I am going to die	1.97	1.20				50.0	5.1	0.60	
Item 7: My children get upset when we talk about the illness	1.88	1.03				46.4	2.1	0.67	
Item 9: My children might be in need of professional mental health care	2.60	1.29				23.5	11.9	0.70	
Item 11: My children get confused or upset by what others say about my illness	1.76	1.00				54.8	1.5	0.61	
**Co-parent**	2.12	0.79	3.00	3.60	4.20				0.81
Item 6: My children’s other parent would not be able to meet their emotional needs if I die	2.12	1.28				44.9	7.4	0.58	
Item 12: There is no one to take good care of my children if I die	2.30	1.38				43.2	8.6	0.60	
Item 13: My partner is not providing me with enough practical support	1.90	1.20				53.9	4.8	0.51	
Item 14: My partner is not providing me with enough emotional support	1.72	1.16				64.3	4.8	0.66	
Item 15: My children’s other parent would not be a responsible caregiver if I die	2.55	1.43				33.3	13.7	0.68	

PCQ: Parenting Concerns Questionnaire; SD: Standard deviation.

### Reliability and item discrimination

The reliability analyses revealed the corrected item-total correlations to be above 0.40, indicating that each item was related to the overall scale. Internal consistency of the subscales was considered adequate with Cronbach’s alpha at 0.85 for practical impact, 0.85 for emotional impact, and 0.81 for the co-parent subscale (Table 2).

### Convergent, criterion, and known-group’s validity

Convergent validity was demonstrated by significant correlations between the PCQ and DASS, FAD-GF, and the GSE scale (Table 3). As anticipated, parenting concerns were positively associated with symptoms of depression, anxiety, and stress (DASS). The PCQ total score and subscales were also positively associated with greater family dysfunction (FAD-GF), with the strongest association observed with the co-parent scale. Greater parenting concerns were associated with lower self-efficacy ratings. Correlations between the three PCQ subscales are also shown in Table 3.

**Table 3:** Correlations between parenting concerns and measures of depression, anxiety, stress, family function, and general self-efficacy (*n* = 336).

Measures	Practical impact scale	Emotional impact scale	Co-parent scale	PCQ total score
Practical impact scale	1	0.68	0.43	0.86
Emotional impact scale	–	1	0.44	0.85
Co-parent scale	–	–	1	0.76
PCQ total score	–	–	–	1
DASS				
Depression	0.44	0.32	0.29	0.43
Anxiety	0.40	0.33	0.25	0.39
Stress	0.51	0.36	0.35	0.50
FAD-GF	0.34	0.30	0.62	0.51
General self-efficacy	-0.28	-0.20	-0.20	-0.28

DASS: Depression, anxiety and stress scale; FAD-GF: the McMaster Family Assessment Device-general functioning scale; PCQ: Parenting concerns questionnaire.

All correlation coefficients significant at *p* < 0.001.

Criterion validity was shown by significant differences in parenting concerns between those who had utilized support services for parental distress compared with those who had not on the practical impact scale (mean = 2.88 [sd = 0.99] vs 2.35 [0.94], Cohen’s *d* = 0.56, *p* < 0.001), the emotional impact scale (2.42 [0.92] vs 2.04 [0.89], Cohen’s *d* = 0.43, *p* < 0.001), the co-parent scale (2.27 [0.97] vs 2.05 [0.98], Cohen’s *d* = 0.22, *p* < 0.001), and on the PCQ total score (2.53 [0.77] vs 2.15 [0.77], Cohen’s *d* = 0.50, *p* < 0.001).

The PCQ detected differences in parenting concerns in relation to cancer status where parents with incurable cancer scored higher than parents with curable cancer on the total scale (mean = 2.48 [sd = 0.82] vs 2.22 [0.78], Cohen’s *d* = 0.33, *p* < 0.05) and the emotional impact scale (2.49 [1.02] vs 2.09 80.88], Cohen’s *d* = 0.45, *p* < 0.05) but not on the practical impact scale (2.74 [0.98] vs 2.47 [0.98], Cohen’s *d* = 0.28, *p* > 0.05) or the co-parent scale (2.21 [0.93] vs 2.10 [0.99], Cohen’s *d* = 0.11, *p* > 0.05).

### Confirmatory factor analysis of the original three-factor model

**Figure 1:**
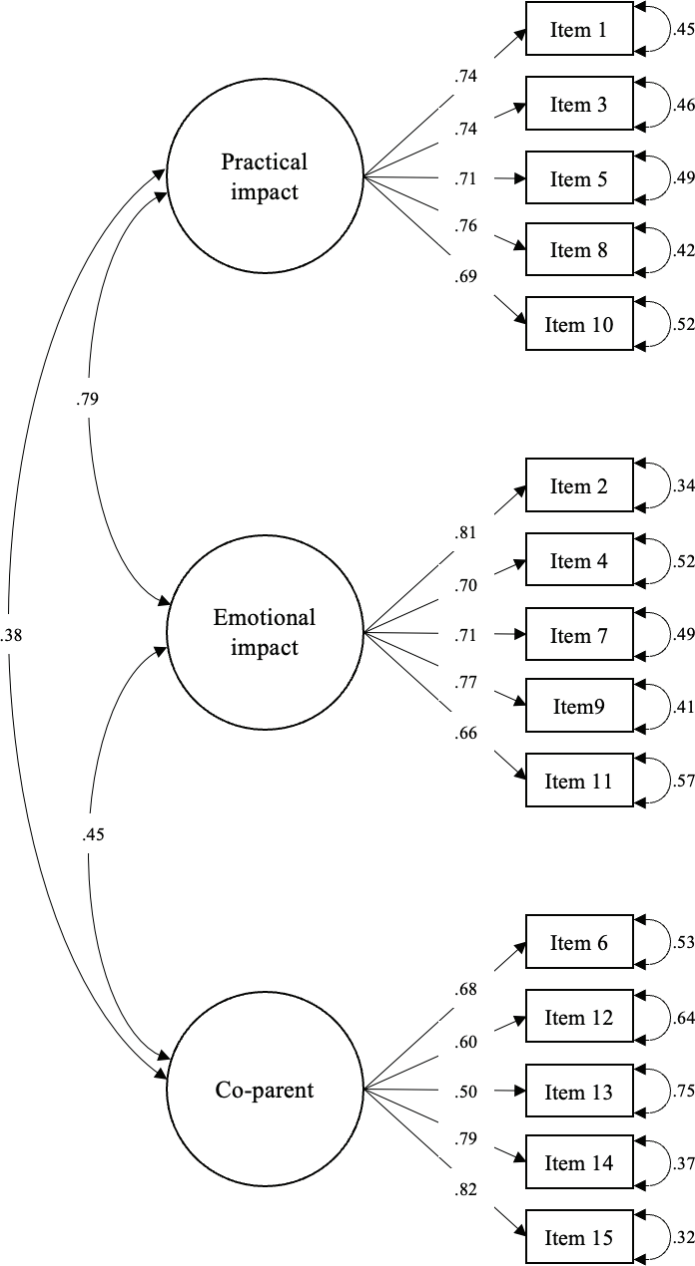
Confirmatory factor analysis of the Parenting Concerns Questionnaire’s original three-factor model (*n* = 336).

Results from the CFA revealed moderate to strong factor loadings for both the practical impact scale (0.69–0.76) and the emotional impact scale (0.66–0.81). However, factor loadings on the co-parent scale ranged from somewhat weaker to stronger (0.50–0.82), with the lowest value observed for item 13 (Figure 1). Positive correlations between the sub-scale factors were observed, with the strongest correlation seen between the practical and emotional impact scales (0.79), followed by emotional impact and the co-parent scales (0.45), and then the practical impact and co-parent scales (0.38). With regard to overall model fit, the significant χ^2^ test (χ^2^(87) = 339.230, χ^2^/*df* = 3.90, *p* < 0.001) and χ^2^/*df* ratio >3, together with a CFI value of 0.89 and RMSEA of 0.093 (90% CI: 0.083–0.103) indicated inadequate fit.

## Discussion

The challenges associated with being a patient with cancer while being a parent of dependent children are under-researched and infrequently addressed in clinical settings despite studies showing the importance of parenting concerns for both parental and child psychological well-being [1–4,12]. The PCQ was developed to explore the specific needs and concerns among parents with cancer and has been shown to be a valid and reliable measure of psychological distress that is specifically associated with parenting in this context [10]. The PCQ has previously been translated and psychometrically evaluated in German and Portuguese parents with cancer [19,20] and in the USA among parents with metastatic cancer [21]. This study examined the psychometric properties of the Swedish version of the PCQ in a sample of patients with cancer who were parents of dependent children.

The results showed that the PCQ had high internal consistency but that the model fit indices indicated an inadequately fitting model for the three-factor structure identified in the original article. As in prior studies, the PCQ demonstrated small-to-moderate correlations in the expected directions, with other measures of psychosocial distress suggesting that the PCQ covers a unique and specific aspect of distress in parents with cancer [10,19–21]. Furthermore, the scales differentiated between those expressing a need for support for parental distress and those who did not, indicating good discriminant validity.

The average score on the PCQ was 2.27, which can be considered as corresponding to parents being ‘somewhat concerned’. This is in line with previous studies, using similar (i.e. online advertising/recruitment) as well as other methods of recruitment including cancer registries and in-clinic recruitment [19–21,30]. However, whereas overall high floor effects on scale items have been observed previously [19], this was not the case in the present study (ranging from 15.2 to 64.3%) suggesting that parenting concerns are indeed not negligible. Items with the largest proportion of ceiling effects observed were concerns related to the practical impact of the parent’s illness on children’s routines, concerns that children may be in need of professional emotional support, and a concern that the co-parent would not be a responsible caregiver should the parent with cancer die. Notably, the sample in the present study included a majority of mothers. One explanation for the discrepancy in parenting concerns may be related to gendered parental roles. Previous studies have underscored that the concerns of mothers and fathers differ, where mothers tend to worry, for example, about the impact cancer has on their children and how to fulfil the maternal role [3,14,31–33], while fathers tend to report more worries regarding financial security and being the provider for the family [31,34]. As such, the gender gap related to parenting stress and concerns may be linked to the persistence of traditional roles regarding the care of children [35].

## Strengths and limitations

There are some limitations that must be taken into consideration when interpreting the findings. We recruited a self-selected sample resulting in a somewhat homogenous sample consisting largely of mothers with breast cancer. Despite the low numbers of fathers in the sample, few previous studies have included fathers at all. As such, we retained the small group of fathers in the study. However, it must be noted that the generalizability of the results is limited. In addition, the data were collected using an online survey tool, and the study invitation was distributed via patient organizations, clinical contacts, and social media networks, which may have excluded participants with less access to online resources or without social media accounts. This method of recruitment may have attracted a sample of parents with particularly high levels of parenting concerns. However, the mean score on the total PCQ was similar to previous studies, including other methods of recruitment [19–21,30].

Whereas adequate model fit has been observed in other settings [19,20], the present results are in line with results from the psychometric evaluation of the PCQ among parents with metastatic cancer in the study by Park et al. [21]. In the present study, 18.5% reported having incurable cancer, which could have influenced the results. Studies observing adequate model fit have included somewhat more heterogenous samples in terms of sex distribution and cancer diagnoses than the present study or the study by Park et al. [21], which may have contributed to differences in model fit. However, model misfit is normal to some degree, and depending on the application context of a scale or questionnaire, exact model fit might be less important [36]. In cases where the accuracy of an individual measurement is of little interest, for example, when group means are compared or predictions of an outside criterion at group level are evaluated, a slightly mis-specified model can still be useful to gain important insights [37].

In previous studies, limitations regarding the co-parent subscale, which not only includes two items that concern partner support rather than parenting specifically but also presumes the presence of a co-parent rather than other adults who may be caring for the children in the absence of a co-parent, have not been addressed. In the present study, we opted to exclude parents who did not have a partner so as to avoid missing data in the analysis. We argue that potential modifications to the measure could include removing the items pertaining specifically to partner support and instead expanding the definition of co-parent to other adults who may care for the children as was done in the PCQ-AD for advanced cancer (‘I am concerned about how other people would raise my child(ren)if I die’) [21].

Revising some items in the PCQ could also potentially better capture the complexity of being a parent with cancer, for example, struggles with communicating about cancer, and incorporating aspects of parenting stress, such as feelings of being overwhelmed by the responsibilities of parenting, especially since parents need to balance dual roles as a parent and a patient. In addition, the fear of missing out and not being there for their children is a worry many parents face that is not addressed in this version of the PCQ. However, increasing the number of items in the scale will also add to the burden on respondents and may not necessarily improve the model fit, as such it is a balancing act. In the co-parent scale, however, items 13 and 14 may benefit from being re-phrased to reflect parenting rather than addressing the partner relationship itself, as was intended; for example ‘My partner is not providing me with enough practical/emotional support *with the child/ren*’ so as to not only capture relationship quality but also how supportive the partner is as a co-parent.

## Conclusion

In conclusion, accurate evaluation of parenting concerns is an important step towards identifying parents with cancer who could benefit from targeted psychosocial interventions. However, the PCQ may require some minor refinement to fully capture the breadth of parenting concerns in different settings. As the original three-factor structure of the PCQ was not fully supported by confirmatory factor analysis in the present study, using the total score on the Swedish version of the PCQ in favour of subscale scores may be advisable.

## Author contributions

AW designed and was PI for the study. LL, MRS, NG, ACM, CWM, and PE contributed to the conception of the work. NG and AW analyzed the data. LL, MRS, NG, ACM, CWM, PE, and AW contributed to the interpretation of data. LL and AW drafted the manuscript, and MRS, NG, ACM, CWM, and PE contributed to revising the manuscript for important intellectual content. All authors have read and approved the final manuscript and agree to be accountable for all aspects of the work in ensuring that questions relating to the accuracy or integrity of any part of the work are appropriately investigated and resolved.

## Data Availability

Data are not publicly available due to ethical restrictions. Data that support the findings of this study are available from the corresponding author upon reasonable request and with necessary ethical approval.
